# Milling byproducts are an economically viable substrate for butanol production using clostridial ABE fermentation

**DOI:** 10.1007/s00253-020-10882-8

**Published:** 2020-09-11

**Authors:** Nils Thieme, Johanna C. Panitz, Claudia Held, Birgit Lewandowski, Wolfgang H. Schwarz, Wolfgang Liebl, Vladimir Zverlov

**Affiliations:** 1 Technical University of Munich, Emil-Ramann-Str. 4, 85354 Freising, Germany; 2 Present Address: Technical University of Munich, Weihenstephaner Berg 3, 85354 Freising, Germany; 3 Present Address: TDK Electronics AG, Rosenheimer Str. 141e, 81671 Munich, Germany; 4 Fritzmeier Umwelttechnik GmbH & Co KG, Dorfstraße 7, 85653 Aying, Germany; 5 Present Address: Electrochaea GmbH, Semmelweisstrasse 3, 82152 Planegg, Germany; 6 Present Address: aspratis GmbH, Huebnerstrasse 11, 80637 Munich, Germany; 7 Institute of Molecular Genetics, RAS, Kurchatov Sq 2, 123128 Moscow, Russia

**Keywords:** Clostridia, Butanol, ABE fermentation, Wheat milling byproducts, Profitability analysis

## Abstract

Butanol is a platform chemical that is utilized in a wide range of industrial products and is considered a suitable replacement or additive to liquid fuels. So far, it is mainly produced through petrochemical routes. Alternative production routes, for example through biorefinery, are under investigation but are currently not at a market competitive level. Possible alternatives, such as acetone-butanol-ethanol (ABE) fermentation by solventogenic clostridia are not market-ready to this day either, because of their low butanol titer and the high costs of feedstocks. Here, we analyzed wheat middlings and wheat red dog, two wheat milling byproducts available in large quantities, as substrates for clostridial ABE fermentation. We could identify ten strains that exhibited good butanol yields on wheat red dog. Two of the best ABE producing strains, *Clostridium beijerinckii* NCIMB 8052 and *Clostridium diolis* DSM 15410, were used to optimize a laboratory-scale fermentation process. In addition, enzymatic pretreatment of both milling byproducts significantly enhanced ABE production rates of the strains *C. beijerinckii* NCIMB 8052 and *C. diolis* DSM 15410. Finally, a profitability analysis was performed for small- to mid-scale ABE fermentation plants that utilize enzymatically pretreated wheat red dog as substrate. The estimations show that such a plant could be commercially successful.

**Key points**

• *Wheat milling byproducts are suitable substrates for clostridial ABE fermentation.*

• *Enzymatic pretreatment of wheat red dog and middlings increases ABE yield.*

• *ABE fermentation plants using wheat red dog as substrate are economically viable.*

**Electronic supplementary material:**

The online version of this article (10.1007/s00253-020-10882-8) contains supplementary material, which is available to authorized users.

**Figure:**
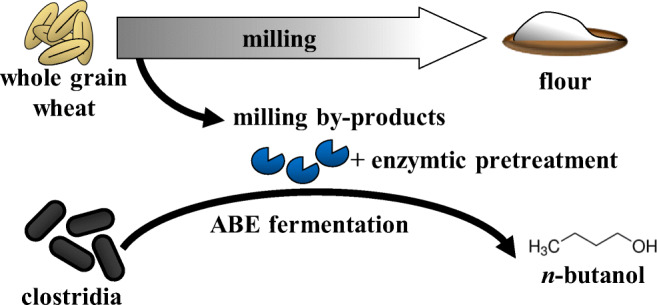

## Introduction

The urgency to shift our current society and industry to more environmentally friendly alternatives has increased in recent years, as more and more studies showed that the resulting global warming will have detrimental effects on nature, human society, and health (Millington et al. 2019; Sun et al. 2019; Ahima 2020). Many possible avenues are explored to mitigate a global crisis, one of them being the replacement of petrochemical processes with sustainable biorefinery approaches to produce chemicals such as *n-*butanol. These processes have a reduced carbon dioxide emission compared with conventional production.

*n-*Butanol is a platform chemical with market sales of around 3.75 to 4.65 billion USD per year (E4tech et al. 2015) and is mainly used in the production of cleaning products, plasticizer, lubricants, coating, and painting additives (National Center for Biotechnology Information 2020). Moreover, *n-*butanol is considered a potential gasoline replacement and fuel additive, superior to ethanol due to its higher energy density, reduced hygroscopy, better pumpability, and compatibility with combustion engines (Dürre 2007; Berezina et al. 2012; Xue et al. 2013). While butanol is mostly produced via a petrochemical route, bio-based butanol accounts for only 20% of the market volume (E4tech et al. 2015).

Bio-butanol can be produced by several solventogenic clostridial species trough acetone-butanol-ethanol (ABE) fermentation (Lee et al. 2008), which was already used on an industrial scale up to the 1960s (Zverlov et al. 2006). In brief, ABE fermentation is a two-stage process, where predominantly acetic and butyric acids are produced during the growth phase, followed by a re-assimilation and transformation of these acids into the corresponding solvents during the stationary phase (Lee et al. 2008). Solventogenic *Clostridium* sp. can utilize the majority of plant-derived mono- and disaccharides, as well as several polysaccharides such as starch and pectin (Berezina et al. 2008; Zhang 2015). Furthermore, some solventogenic clostridia are able to depolymerize hemicelluloses such as xylan, but none is known to degrade cellulose (Lee et al. 2008; Berezina et al. 2012). However, large-scale commercial production of bio-butanol by clostridia on traditional feedstocks such as corn, sugar, and starch is prevented by high substrate cost as well as competition with food production (Qureshi and Blaschek 2001a; Gu et al. 2011). For example, more than 5.4 t of corn has to be fermented to receive one ton of butanol (Karimi et al. 2015). Therefore, cheaper feedstocks need to be made available for a sustainable and economically profitable production of butanol.

One feasible substrate source for fermentative ABE production could be milling byproducts. Unlike seasonal substrates, such as corn, they are available all year round and constitute ~ 25 to 30% of milling products (Huang et al. 2014). In Bavaria (Germany), around ~ 250 kt of wheat milling byproducts are produced each year, of which ~ 45 kt are wheat red dog, while ~ 90 kt are wheat brans, including wheat middlings (Bayerische Landesanstalt für Landwirtschaft 2012). Wheat milling byproducts are mainly used as animal feed, because they are rich in amino acids, phosphorus, and starch (Huang et al. 2014). However, their relatively low lignin and cellulose content combined with a significant amount of starch and hemicelluloses makes them also suitable substrates for clostridial ABE fermentation (Vogel 2008; Bayerische Landesanstalt für Landwirtschaft 2012; Huang et al. 2014). Furthermore, with their broad availability and low prices, wheat milling byproducts could reduce substrate costs significantly (proplanta.de 2020).

In the present study, a bacterial strain collection of clostridial type strains and newly isolated wild strains was screened for ABE fermentation utilizing wheat milling byproducts as substrate. The fermentation process of the two most promising strains was optimized to further enhance their ABE production, including enzymatic substrate pretreatment. Finally, a profitability analysis of small- to mid-scale ABE fermentation plants utilizing wheat red dog as substrate was calculated. Our estimations show that the strains identified in this study and wheat red dog could be used to produce butanol on an industrial scale at a profitable level.

## Methods

### Clostridia strains and cultivation

The *Clostridium* strains used in this study were from a strain collection gathered at the Chair of Microbiology (Technical University of Munich, TUM) and were screened for ABE production on wheat milling byproducts. Table 1 contains a list of used strains and their species affiliation, as determined by sequencing the 16S rRNA gene. Strains were stored as spore suspensions.

**Table 1:** Screening of clostridial strains for ABE yield on wheat red dog. The strains were incubated for 7 days on GM plus 7.2% (w/v) wheat red dog. ABE yield was determined in culture supernatant by GC. Only strains that reached a minimum butanol yield of 6 g/L are presented

		Solvents in g/L			
Short ID	Organism	Acetone	Ethanol	Butanol	Total
002	*C. beijerinckii* NCIMB 8052	2.87	0.10	8.19	11.16
006	*C. saccharobutylicum* DSM 13864	3.81	0.21	6.55	10.57
007	*C. saccharoperbutylacetonicum* DSM 14923	2.61	0.12	8.56	11.29
041	*C. saccharobutylicum* NCP262	3.86	0.17	6.13	10.17
123	*C. beijerinckii*^†^	2.72	0.87	6.00	9.58
125	*C. saccharobutylicum*^†^	0.09	3.72	7.86	11.67
126	*C. beijerinckii*^†^	0.06	3.93	6.79	10.78
127	*C. beijerinckii*^†^	2.40	0.15	7.91	10.46
129	*C. diolis* DSM 15410	2.51	0.11	7.80	10.42
131	*C. saccharoperbutylacetonicum*^†^	4.85	0.26	6.91	12.03

^†^Wild strain isolated from Chair of Microbiology (TUM)

Strains were incubated in 100-ml butyl rubber-stoppered serum bottles that were made anaerobic through vacuum and replacement of air with 98% N_2_ + 2% H_2_, followed by autoclaving (Rettenmaier et al. 2019). *Grundmedium* YAF25 (GM), a medium specifically optimized for solventogenic clostridia, was used for most experiments. GM was composed of 5 g (w/v) yeast extract (Carl Roth), 65 mM ammonium acetate (Carl Roth), and 90 μM iron (II) sulfate heptahydrate (Carl Roth) dissolved in 1-L tap water. The pH of the medium was adjusted to 6.8. Alternatively, the strains were incubated in anaerobic tap water (Freising) supplemented with 90 μM iron (II) sulfate heptahydrate (Merck). Spore suspensions of the strains were activated at 60 °C for 10 min and diluted 1:100 in GM with 5% (w/v) sterile-filtered d-glucose (Merck). Initial spore activation was followed by two rounds of pre-cultivation in GM for 48 h at 34 °C. Pre-cultures were inoculated 1:50 in GM plus either wheat red dog or wheat middlings (provided by the Bayerischer Müllerbund e.V., Munich, Bavaria, Germany). The amount of substrate is listed in the respective experiments. The strains were incubated for up to 7 days. Culture supernatant samples were taken every 24 h for gas chromatography (GC), as described in the respective experiments.

### Gas chromatography

The concentrations of acetone, ethanol, and *n-*butanol were determined using Nexis GC 2010 or Nexis GC 2030 systems (Shimadzu), as described previously (Koeck et al. 2015). In brief, the samples were diluted 1:5 with distilled water at pH 2 (with hydrochloric acid) and 0.05% (w/w) 1-propanol was added as an internal standard. Ten microliters of culture samples was injected at 250 °C to a 65 m Restek Stabilwax®-DA column with an inner diameter (ID) of 0.32 mm and film thickness of 0.5 μm. A temperature gradient of 6 °C/min from 50 to 80 °C and 25 °C/min from 80 to 250 °C was used during elution. Flame ionization detection (FID) at 260 °C was used to measure the eluted compounds.

### Enzymatic digests of substrates

The commercially available cellulase mixtures AlternaFuel-200P (Dyadic International Inc.), Accellerase XC (GENENCOR), and Cellic CTec2 (Merck) were used to pretreat wheat middlings. The pretreatment was performed at 34 °C and 50 °C in either GM or a buffer containing 1 M MES pH 5 and 0.1 M calcium chloride. Samples were taken after 24 h and 48 h of incubation (enzymes in GM) or after 4 h and 24 h (enzymes in buffer). Released sugars were determined by thin layer chromatography (TLC), as described previously (Baudrexl et al. 2019), and DNSA method (Miller 1959) to determine reducing ends.

### Profitability analysis

The profitability analysis was performed by Fritzmeier Umwelttechnik GmbH & Co KG (Großhelfendorf, Germany). Several economic scenarios were calculated for either wheat red dog or wheat middlings as substrate. The most profitable scenarios are presented in this study. The prices, values, and other factors influencing the analyses were based on data from Bavaria (Germany). Initial expenditures, such as acquiring the building lot, building the process facilities, and infrastructure, were not calculated as they are only one-time expenditures and subject to change. Moreover, a complete recirculation of process water was deemed necessary for the whole process to eliminate the cost of water during the fermentation process. Post-processing of the solvents, e.g., distillation and purification, were also not considered in these calculations. The continuous development in this field leads to more economical butanol extraction methods, requiring specialized equipment and chemicals (see e.g. Kujawska et al. 2015; Segovia-Hernández et al. 2020). Furthermore, the fermentation plant was designed as semi-continuous processing plant, based on published data for optimized ABE production using plant biomass as substrate as was described in Zverlov et al. (2006). The calculations regarding the profitability analysis are the intellectual property of Fritzmeier Umwelttechnik GmbH & Co KG.

## Results

### Identification of strains with high butanol yield on wheat milling byproducts

The collection of bacterial strains from the Chair of Microbiology (TUM), which is composed of type strains as well as newly isolated wild strains, was screened for ABE fermenting clostridia strains that are suitable for consolidated bioprocessing of milling byproducts (Table 1). A total of 30 strains were tested for ABE fermentation in *Grundmedium* YAF25 (GM) plus either 7.2% wheat red dog or 13% wheat middlings as carbon source (data not shown). These substrate amounts equaled ~ 5% (w/v) of soluble sugars and starch in the medium (Bayerische Landesanstalt für Landwirtschaft 2012). Furthermore, by using milling byproducts as a substrate, it should be ensured that the tested strains are able to grow on complex plant materials and can produce sufficient butanol for industry purposes. Since the used substrates are insoluble and have a high optical density (Figure S1), the growth of the tested strains was approximated through their ABE fermentation yields. Only ten strains exhibited a butanol yield of at least 6 g/L on wheat red dog (Table 1), which we deemed the minimum yield for industrial purposes. The strains did not reach this threshold when incubated on wheat middlings and produced only negligible quantities of either acetone or ethanol (Table S1).

### Optimization of fermentation conditions

**Fig. 1:**
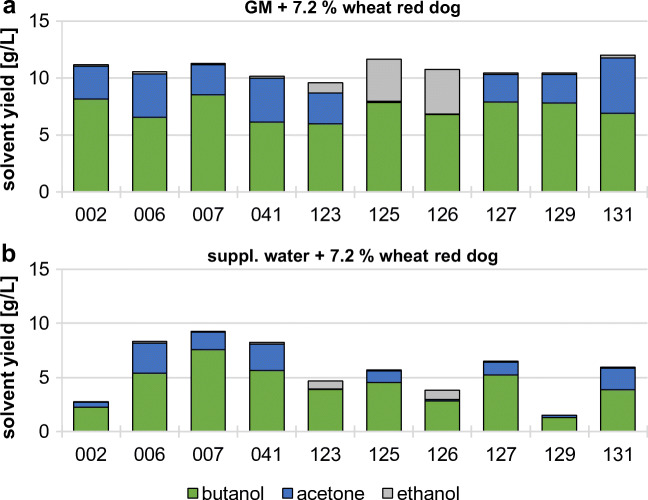
Solvent production of clostridia strains on different media. The strains were incubated for 7 days at 34 °C in either GM (**a**) or tap water (**b**) supplemented with Fe(II)SO_4_. 7.2% (w/v) wheat red dog was used as a carbon source in both media. Solvent yield in the culture supernatant was determined using GC

Since the substrates used in this study are complex carbon sources, with high protein, fat, fiber, and mineral contents (Bayerische Landesanstalt für Landwirtschaft 2012), we hypothesized that a reduced medium consisting of only tap water might be sufficient to allow ABE fermentation of the *Clostridium* strains. To test this hypothesis, we used the ten strains that showed the highest butanol yield on wheat red dog and incubated them for 7 days in either GM or Fe(II)SO_4_ supplemented tap water (Fig. 1). In both cases, 7.2% (w/v) wheat red dog was used as a substrate. While the ten tested strains performed adequately on GM plus wheat red dog, reaching butanol yields of up to 8.2 g/L (Fig. 1a), the majority of strains were not able to produce suitable amounts of butanol when incubated in supplemented tap water (Fig. 1b). Only the four strains 006, 007, 041, and 127 reached a butanol yield of > 5 g/L (5.4 g/L, 7.6 g/L, 5.6 g/L, and 5.2 g/L, respectively), only ~ 80% or less of the total ABE yield compared with GM. Therefore, we decided to use GM as a medium for the following optimization experiments.

**Fig. 2:**
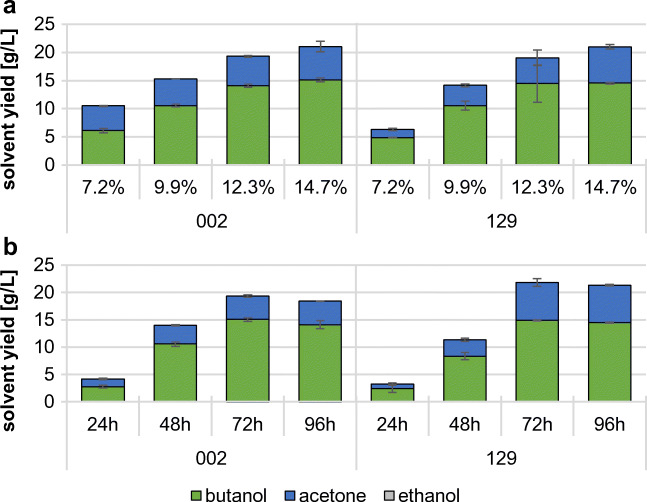
Determination of best-suited fermentation settings. Solvent yield in culture supernatant was determined using GC. **a** The strains 002 and 129 were incubated for 96 h at 34 °C in GM plus different concentrations of wheat red dog. **b** The strains were incubated at 34 °C for 96 h in GM plus 14.7% wheat red dog. Samples of the culture supernatant were taken every 24 h. Error bars represent standard deviation (*N* = 2)

The strains 002 and 129 were used to further optimize the fermentation conditions with wheat red dog as a substrate, because they exhibited the most promising butanol yields on this milling byproduct (8.2 g/L and 7.8 g/L, respectively) (Fig. 1a). Initially, the strains were grown for 96 h on different amounts of wheat red dog. Based on the starch and free sugar content of the substrates (Table 2), we decided to determine the ABE yield of both strains after incubation on 7.2%, 9.9%, 12.3%, and 14.7% wheat red dog (Figure S1). Higher substrate concentrations could not be tested, due to the extremely high viscosity of the milling byproducts in water, which prevents the utilization of more than about 15% (w/v) in batch fermentations (data not shown). The experiment showed that both strains exhibited their best butanol and overall solvent production when 14.7% wheat red dog (equaling 11% of soluble sugars and starch content) were used as substrate (Fig. 2a). Butanol yields of ~ 15 g/L and ~ 14.5 g/L were determined for the strains 002 and 129, respectively. Based on these observations, we determined the time needed for optimum ABE yield. The strains 002 and 129 were incubated over a period of 96 h in GM plus 14.7% wheat red dog. Samples were taken every 24 h and their ABE content was determined by GC (Fig. 2b). The butanol concentration increased steadily over time and reached its peak at 72 h at ~ 15 g/L for both strains. Therefore, a fermentation period of 72 h while using ~ 15% (w/v) of the substrate was deemed the best setup for commercial ABE fermentation of milling byproducts.

**Table 2:** Substrate composition of wheat middlings and wheat red dog. Data determined by Bayerische Landesanstalt für Landwirtschaft 2012

Content	Wheat middlings	Wheat red dog
Dry matter	892 g	875 g
Ash	4.2%	1.6%
Protein	17.4%	16.7%
Lipids	4.3%	3.0%
Fiber	7.7%	0.9%
Starch	32.5%	72.1%
Soluble sugars	4.9%	3.5%

### Enzymatic pretreatment of milling byproducts

**Fig. 3:**
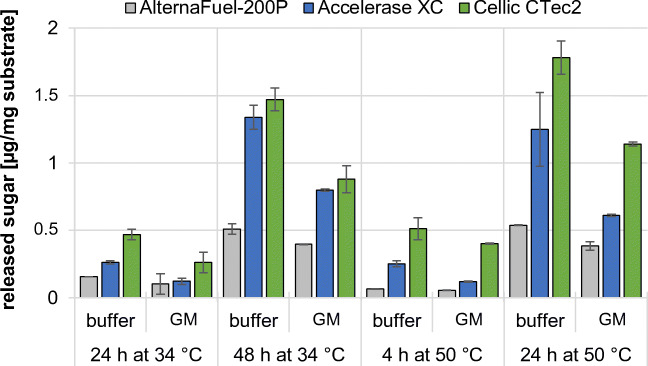
Released sugars after enzymatic pretreatment of wheat middlings. Three commercially available enzyme mixtures were used to macerate 1% wheat middlings. The enzymes were tested in their respective buffer or GM. Incubation at 34 °C was performed for 24 h and 48 h (fermentation temperature), while the enzyme mixtures were also incubated at 50 °C (optimal temperature) for 4 h or 24 h. The released reducing end containing sugars were determined by DNSA assay. Error bars represent standard deviation (*N* = 2)

Another possible avenue for increased ABE yield of clostridia is substrate pretreatment (Kumar et al. 2009). Here, we decided to test enzymatic hydrolysis of the substrate to increase the fermentation performance. We tested three commercially available cellulase mixtures, AlternaFuel-200P, Accelerase XC, and Cellic Ctec2, either at their optimal reaction-temperature of 50 °C (as indicated by the manufacturer) or at 34 °C, the temperature during fermentation. Moreover, the activity of the three enzyme mixtures was tested in either buffer or GM (Fig. 3). Wheat middlings were used as a substrate for enzymatic pretreatment, since the strains exhibited a reduced ABE production on this substrate compared with wheat red dog (Table S1), possibly due to the higher fiber content, including cellulose as well as hemicellulose compounds such as xylan and ß-glucan. The released sugars were determined by reducing the end assay (DNSA).

The enzyme mixtures were able to liberate sugars under all conditions tested (Fig. 3). However, the best enzymatic performance was achieved by using buffer, while GM led to the weakest release of sugars (~ 66% of activity in buffer). Furthermore, the enzymatic pretreatment was executed over different durations. At 34 °C, the incubation was performed for up to 48 h, while samples incubated at 50 °C were measured after 4 h and 24 h. However, even after 48 h, the released sugars at 34 °C were less compared with 24 h at 50 °C. Besides the conditions, the enzyme mixtures showed different release rates of sugars. AlternaFuel-200P exhibited the lowest release of sugars in each condition, while Accelerase XC and Cellic CTec2 performed similarly in buffer. Still, Cellic CTec2 released the most soluble sugars in all conditions. These results show that a 24-h incubation of the substrate at 50 °C is the best-suited pretreatment of the substrate.

To identify the sugar moieties released during enzymatic digest, the supernatants of the enzyme assays performed at 50 °C for 24 h were analyzed using thin-layer chromatography (TLC). Xylo-oligosaccharides, xylose, and glucose were used as reference substances, while buffer without substrate was used as a negative control (Figure S2). AlternaFuel-200P yielded only faint spots for glucose and some xylo-oligosaccharides. In contrast, Accelerase XC and Cellic CTec2 treatment led to strong spots with mobilities corresponding to glucose and xylotriose. Cellic CTec2 also yielded a strong spot corresponding to xylose. Therefore, Cellic CTec2 leads to a better reduction of the hemicellulose fraction of wheat middlings to xylose.

**Fig. 4:**
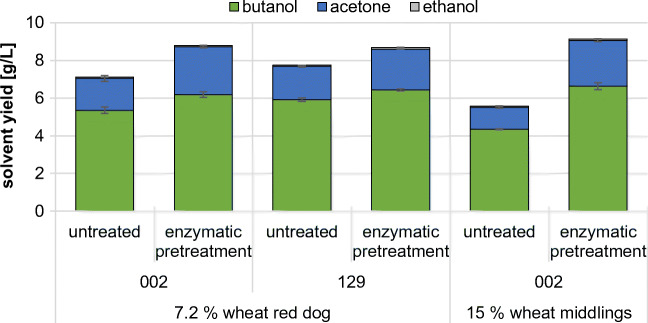
ABE yield of clostridia on enzymatically pretreated substrate. The strains were incubated for 72 h on GM plus either 7.2% wheat red dog or 15% wheat middlings as carbon source. The substrate was treated with Cellic CTec2 for 24 h at 50 °C prior to fermentation. Untreated substrate was used as control. Solvent yield in the culture supernatant was determined using GC. Error bars represent standard deviation (*N* = 2)

To analyze whether the enzymatic pretreatment of milling byproducts leads to increased ABE yields, 7.2% wheat red dog and 15% wheat middlings were enzymatically macerated for 24 h at 50 °C using Cellic CTec2. The amounts of substrates were chosen to equal 5% soluble sugars and starch. After pretreatment, the milling byproducts were used as a carbon source in GM and the strains 002 and 129 were incubated on wheat red dog, while only strain 002 was incubated on wheat middlings. All conditions were compared with the untreated substrates as controls (Fig. 4). All tested strains exhibited improved ABE yields on both wheat red dog and wheat middlings. While the butanol production of the strains 002 and 129 was increased by ~ 9% and ~ 15% on wheat red dog, respectively, the butanol production of strain 002 could be improved by ~ 53% on wheat middlings (Fig. 4). These results indicate that even less suited substrates, i.e., wheat middlings, can lead to suitable butanol yields after enzymatic pretreatment.

### Profitability analysis of wheat milling byproducts as substrates for clostridial ABE fermentation

This study showed that milling byproducts can be efficiently utilized by solventogenic clostridia to produce considerable amounts of butanol. To analyze if such an approach would be economical feasible, a profitability analysis of a hypothetical ABE fermentation plant located in Bavaria (Germany) was performed. This plant would use wheat milling byproducts as a substrate for fermentation as well as the two best ABE forming clostridiastrains 002 and 129 as production organisms.

To calculate the costs of material, machinery, and additional expenses, previous publications describing fermentation plants and process facilities of similar size were used (Gapes 2000; Qureshi and Blaschek 2001a, b). As there are no prices for wheat red dog available at the market, the price for wheat middlings was used. This price can change quite significantly over the course of a year and between years. However, the calculations were based on a fixed price of 150 €/t substrate (the approximate annual average of 2019). The two scenarios of either 7000 t/a or 35,000 t/a substrate were chosen due to the amount of substrates available. Wheat red dog is produced all year round at a quantity of 45,000 t/a, while 90,000 t of wheat middlings are produced each year (Bayerische Landesanstalt für Landwirtschaft 2012). Transportation costs were estimated to be 10 €/t of substrate, if the transport distance remains below 100 km.

The contamination of semi-continuous fermentation processes by bacteria or phages poses a significant problem (Zverlov et al. 2006). However, the impact of these contaminations can be reduced by thermal pretreatment of the substrates and application of a high cleanliness standard. This led to the inclusion of autoclaves and clean-in-place/sterilization-in-place (CIP/SIP) processes into the initial investment calculation (Table 3).

**Table 3:** Overview of initial and annual costs of an ABE fermentation plant using wheat red dog as a substrate. Two scenarios with either 7000 t/a or 35,000 t/a of utilized substrate are presented. The expenses are calculated with an uncertainty factor of 25%. *CIP/SIP*, clean-in-place/sterilization-in-place

	Amount of wheat red dog	
Expenses	7000 t/a	35,000 t/a
Investment costs (initial) • Fermenter (pre-culture) • Fermenter (main culture) • Filtration facility • Stripping facility • Distillation facility • Adsorption/desorption • CIP/SIP processes • Autoclave • Assembly • Pipework/instruments • Electrics	1,237,500 €	4,950,915 €
Capital costs (annual) • Investment in machines/tools • Investment in distribution facilities • Interest payments	173,250 €/a	693,128 €/a
Overhead (annual) • Staff • Insurances	198,094 €/a	402,377 €/a
Operation costs (annual) • Electricity • Heating • Supplies and equipment • Maintenance (equipment) • Maintenance (building)	92,813 €/a	371,319 €/a
Process costs (annual) • Substrate • Transportation • Enzymatic hydrolysis	1,122,460 €/a	3,863,145 €/a
Total (annual)	1,586,616 €/a	5,329,969 €/a

Furthermore, various pretreatments of the substrates were considered for different scenarios. Based on internal calculations, an enzymatic pretreatment of the substrate with Cellic CTec2 would cost 2544 €/a for 7000 t/a substrate or 12,723 €/a for 35,000 t/a substrate. In contrast, a weak acidic hydrolysis of the substrate would account for around ~ 1,000,000 €/a or ~ 5,400,000 €/a for 7000 t/a or 35,000 t/a substrate, respectively.

Based on the gathered data, the initial and annual expense of an ABE fermentation plant utilizing wheat millings were calculated (Table 3). Calculations with wheat middlings as the substrate led to an annual net loss of several thousand Euros even under the best circumstances (data not shown) and were therefore not further analyzed as potential substrate.

**Fig. 5:**
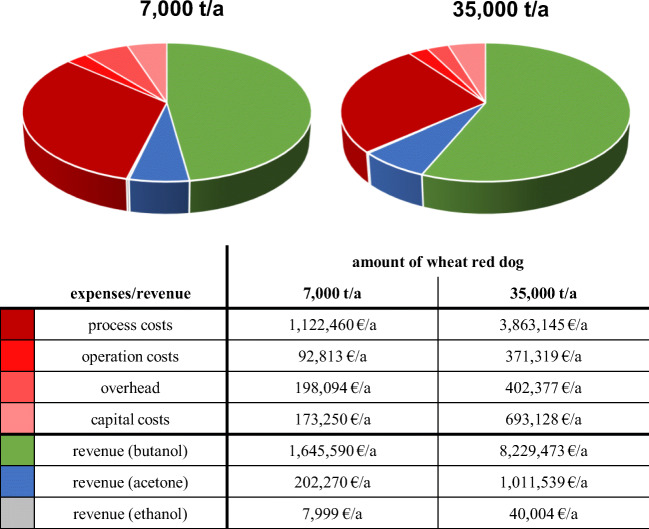
Profitability calculation of ABE fermentation plants using wheat red dog as substrate. Only annual costs and revenues are presented in the charts and associated table

To calculate proceeds made by the hypothetical fermentation plant, the strain 002 was considered the best-suited candidate for commercial ABE fermentation of wheat red dog. Strain 002 exhibited total solvent production rates of ~ 20 g/L when incubated on 14.7% wheat red dog (Fig. 2a) and had an ABE ratio of 3:7:0 (acetone:butanol:ethanol). An increased ABE yield of up to 20% was assumed for strain 002 when using enzymatically pretreated material as a substrate. The product revenues for butanol, acetone, and ethanol were estimated based on previously published data (Qureshi and Blaschek 2001a, b) as well as market prices. Product revenues of 2000 €/t, 600 €/t, and 700 €/t were calculated for butanol, acetone, and ethanol, respectively. These prices are dependent on the global economic situation and correlated to the oil price, among other things. A scenario with a high oil price and equally high ABE prices was chosen for this profitability analysis. Similar prices were observed over a 10-year period from 2003 to 2013. Although the market price for butanol has changed in recent years, the fermentation plant would remain profitable with a low price of ~ 1100 € per ton of butanol. Taken together, annual revenues of up to 1,855,860 € or 9,281,017 € can be achieved by selling the solvents produced on either 7000 t or 35,000 t wheat red dog (Fig. 5), respectively. The calculation presented here does not factor in revenues made by selling the gas produced by the strains. The proceeds of ABE fermentation plants using either 7000 t/a or 35,000 t/a wheat red dog would be 269,244 €/a or 3,951,048 €/a, respectively. These values can be further increased through improved bioprocesses, e.g., gas-stripping for maximized solvent yield (Ezeji et al. 2007).

## Discussion

In this study, wheat red dog and wheat middlings were analyzed as a substrate for industrial-scale ABE fermentation. Both substrates are produced throughout the whole year in large quantities and they are of low economic value (Huang et al. 2014). Moreover, most wheat milling byproducts are rich in starch and hemicelluloses (Bayerische Landesanstalt für Landwirtschaft 2012), which can be utilized by some clostridial strains as a carbon source during ABE fermentation (Berezina et al. 2008).

At first, we had to identify clostridia strains that can utilize wheat red dog or wheat middlings as carbon source and produce suitable amounts of butanol during fermentation. A collection of type strains as well as wild isolate strains was screened for clostridia that exhibited a butanol yield of > 6 g/L. Ten strains could be identified that met this threshold on wheat red dog, while none of the strains was able to produce sufficient amounts of butanol on wheat middlings. The observed differences in ABE quantities between the two substrates might be caused by their composition. Although the amount of free soluble sugar and starch were set to be identical with both substrates (~ 5%), the overall quantity of added substrate differed greatly (7.2% of wheat red dog vs. 13% of wheat middlings). Wheat middlings have almost nine times the amount of fiber than wheat red dog, while having less than ~ 50% of its starch content (Table 2). Therefore, the starch in wheat middlings might not be as readily available for hydrolytic enzymes as in wheat red dog. This could impede bacterial growth and metabolite formation by restricting the amount of available carbon source.

Another factor that might have influenced the fermentation rates in this study was the pH of the batch cultures. We performed batch culture fermentations without pH control. High concentrations of readily available carbon sources, such as glucose, can lead to excessive acid production, which in turn can prevent the switch to the solventogenic phase of the cultured clostridia (Maddox et al. 2000). It is feasible that such an “acid crash” could have occurred in some cultures incubated on wheat red dog, since this substrate is starch- and sugar-rich. In addition, while most industrially used clostridia are able to at least partially utilize material rich in hemicellulose and starch (Zverlov et al. 2006; Antoni et al. 2007; Xin et al. 2017; Al-Shorgani et al. 2019), not all of them are able to do so (Berezina et al. 2008). Furthermore, some mesophilic clostridial strains have gene clusters for cellulosomes, complex multi-protein machineries that are used to degrade cellulose (Bayer et al. 2004). Recently, putative cellulosomal genes were identified in three mesophilic *Clostridium* species, including *Clostridium acetobutylicum* and *Clostridium saccharoperbutylacetonicum* (Dassa et al. 2017). However, only the cellulosome of *C. saccharoperbutylacetonicum* N1-4 (HMT) appears to be actively expressed, although the organism was still not able to utilize purified cellulose as substrate (Levi Hevroni et al. 2020). Levi Hevroni et al. hypothesize that cellulosome expression in *C. saccharoperbutylacetonicum* might be dependent on specific inducers which can only be found in complex substrates, such as plant biomasses (Montoya et al. 2001; Levi Hevroni et al. 2020). While we did not examine the cellulose utilization of our strains in this study, we would like to speculate that at least some of them might be able to use a small part of the cellulose content of our complex substrates as a carbon source.

Wheat milling byproducts are complex biomasses that contain most compounds and substances necessary for bacterial growth, such as polysaccharides, soluble sugars, proteins, and trace elements (Bayerische Landesanstalt für Landwirtschaft 2012). Therefore, we hypothesized that Fe(II)SO_4_-supplemented tap water might be a cost-efficient alternative for standard growth media during fermentation when using wheat milling byproducts as a substrate. However, the ABE yields of our tested strains were significantly reduced on supplemented tap water with wheat red dog as a carbon source compared with using GM as a medium. It is possible that crucial trace elements and amino acids present in the complex substrates need further liberation from the biomass during the fermentation, since they are bound in the plant cells (Makino and Osmond 1991; Keegstra 2010; Muñoz-Huerta et al. 2013). These additional steps could have slowed down the whole process and, therefore, reduced the ABE yield. In contrast, GM is rich in available amino acids, trace elements, and nitrogen sources. Furthermore, GM contains 65 mM ammonium acetate, which should allow an easier shift of the pH balance towards the acidic and should lead to an earlier switch from acetogenesis to solventogenesis in clostridia (Grupe and Gottschalk 1992). Taken together, supplemented tap water could reduce the process cost during fermentation and might be an alternative to complex growth media, but at the cost of a curtailed ABE yield.

The strains 002 and 129 exhibited high butanol yields of up to ~ 8 g/L during our initial screenings. Strain 002 is *C. beijerinckii* NCIMB 8052, a well-established ABE production strain (Zhang et al. 2012; Wen et al. 2014), while strain 129 is *C. diolis* DSM 15410, which also showed the potential for high butanol yields in previous studies (Wang et al. 2013). These strains were used to further optimize the fermentation conditions on wheat red dog. We determined that 72 h is the time point for the highest butanol yield during batch fermentation of wheat red dog. Similar time periods for optimal butanol yields were observed for *C. acetobutylicum*. In pH-optimized batch fermentation of *C. acetobutylicum* on glucose and sulfuric acid–hydrolyzed de-oiled rice bran, the peak butanol production was determined after about 72-h incubation (Maddox et al. 2000; Al-Shorgani et al. 2019). However, soluble sugars were used as a substrate in both of these studies, while we used complex biomasses in our study. In addition, these experiments showed that the amount of produced butanol correlated with the increasing concentrations of starch and soluble sugars in the medium. The highest possible amount of wheat red dog (14.7%) led to the best butanol yields of up to 15 g/L for both tested strains. A substrate amount of 14.7% wheat red dog equals ~ 11% soluble sugar and starch content, which is more carbon source than is usually supplied during batch fermentations with clostridia. However, starch as a polysaccharide requires the production of enzymes for degradation. Therefore, the glucose moieties bound in starch are not readily available for consumption, which might delay ABE production. The large amounts of starch used in the fermentations of this study might have compensated these delays, leading to improved ABE production.

Another avenue to increase butanol production is substrate pretreatment. While dilute acid pretreatments are effective at macerating plant material and liberating soluble sugars usable for fermentation (Kumar et al. 2009; Al-Shorgani et al. 2019), these processes are cost-intensive and prolong the whole process significantly (Wyman et al. 2005). However, enzymatic pretreatment of lignocellulosic substrates is an alternative able to increase the soluble sugars available for fermentation in the broth (Kumar et al. 2009; Hosseini Koupaie et al. 2019). In this study, three commercially available (hemi-)cellulase mixtures were tested to improve ABE fermentation yields. Of the three tested enzyme mixtures, Cellic CTec2 showed the most promising results by releasing glucose and xylose from our wheat middlings during incubation. The increased release of easily metabolizable monosaccharides by Cellic CTec2 could be advantageous for improved growth of the clostridia and enhanced ABE production of the strains. This assumption was later corroborated by fermentation experiments of the strain 002 on Cellic Ctec2-pretreated wheat red dog and wheat middlings. The readily available monosaccharides released by the enzyme mixture most likely enhanced the growth rate of the strains on the substrate. Moreover, Cellic CTec2 releases xylose besides glucose, allowing the strains to utilize multiple monosaccharides in parallel. Although clostridia prefer glucose over xylose in published literature (Ounine et al. 1985; Aduse-Opoku and Mitchell 1988), non-diauxic utilization of glucose and other monosaccharides is present in some prokaryotic and eukaryotic species (Hudson et al. 1990; Protzko et al. 2019).

Based on these experimental data, a profitability analysis of a hypothetical small (7000 t/a substrate)- to mid-scale (35,000 t/a substrate) fermentation plant was performed. The plant was located in Bavaria (Germany), because this region has a high density of small mills in close proximity to each other (Bundesinformationszentrum Landwirtschaft 2019). This limits the transport distance of the substrates to below 100 km and makes transport of the materials affordable and more environmentally friendly. Moreover, the substrate was shown to be available in sufficient amounts (Bayerische Landesanstalt für Landwirtschaft 2012). The plant would produce enough revenue to be profitable, if enzymatically pretreated wheat red dog was used as substrate. Further optimizations, for example the utilization of genetically engineered fermentation strains such as *C. beijerinckii* BA101 (Qureshi and Blaschek 2001a, b) or co-fermentation with cellulolytic clostridia (Xin et al. 2017), could significantly increase the butanol yield and, in turn, the revenue of the fermentation plant. A cost analysis of Al-Shorgani et al. in 2019 also demonstrated that another agricultural byproduct, de-oiled rice bran, could be used as substrate for an economically viable ABE fermentation plant (Al-Shorgani et al. 2019). However, the de-oiled rice bran was treated with dilute acids to gain a hydrolysate and the fermentation process was designed to be continuous. In contrast, the fermentation plant designed in the here-presented study would work in a semi-continuous process. This would allow the use of wheat red dog as a non-soluble substrate, providing the full range of substances and compounds in this plant material for the fermentation process.

Overall, the profitability analysis showed that utilizing milling byproducts such as wheat red dog for ABE fermentation can be a suitable and economically viable basis for the industrial production of butanol. However, the data in this study also suggest that the selection of a suitable fermentation strain, such as 002 from this study, has a significant impact on the success of an ABE fermentation plant. Not all strains are suited for utilizing complex plant biomasses as substrates and their ABE yields significantly differ depending on the carbon source provided. Furthermore, the success of a commercial ABE fermentation plant depends on the provided carbon source. While both wheat red dog and wheat middlings are milling byproducts, they differ drastically in their composition. The starch-rich wheat red dog can be used with minor substrate pretreatment to achieve sufficient solvent yields. In contrast, additional pre- and post-processing methods have to be applied to the biorefinery to generate the required ABE yields, if wheat middlings are used as a substrate.

## Electronic supplementary material

ESM 1 (PDF 356 kb)

## Data Availability

All data generated during this study are included in this article or its supplementary information files. Raw datasets are available from the corresponding authors on reasonable request.
