# Supplementary material for: Milling byproducts are an economically viable substrate for butanol production using clostridial ABE fermentation
Source: Appl Microbiol Biotechnol. 2020 Sep 11;104(20):8679–89. doi: 10.1007/s00253-020-10882-8 (PMC7502454; doi:10.1007/s00253-020-10882-8)

Journal name: Applied Microbiology and Biotechnology

Manuscript title: Milling byproducts are an economically viable substrate for butanol production using clostridial ABE fermentation

Authors names: Nils Thieme[1,a], Johanna C. Panitz[1,4,a], Claudia Held[1,5], Birgit Lewandowski[2,6], Wolfgang H. Schwarz[1,7], Wolfgang Liebl[1], Vladimir Zverlov[1,3†]

Authors affiliation(s) and address(es):

[1] Chair of Microbiology, Technical University of Munich, Emil-Ramann-Str. 4, 85354 Freising, Germany

[2] Fritzmeier Umwelttechnik GmbH & Co KG, Dorfstraße 7, 85653 Großhelfendorf, Germany

[3] Institute of Molecular Genetics, RAS, Kurchatov Sq 2, 123128 Moscow, Russia

[4] current address: Chair of Biopolymer Chemistry, Technical University of Munich, Weihenstephaner Berg 3, 85354 Freising, Germany

[5] current address: TDK Electronics AG, Rosenheimer Str. 141e, 81671 Munich, Germany

[6] current address: Electrochaea GmbH, Semmelweisstrasse 3, 82152 Planegg, Germany

[7] current address: aspratis GmbH, Huebnerstrasse 11, 80637 Munich, Germany

[a] authors contributed equally

[†] corresponding author

Corresponding author:

Vladimir Zverlov

E-mail address: vladimir.zverlov@tum.de

Telephone number: +49 8161 / 71 5474

Fax number: +49 8161 / 71 5475

Table S1: ABE yield of clostridial strains on wheat middlings. The strains were incubated for 7 d on GM plus 13 % (w/v) wheat middlings. ABE yield was determined in culture supernatant by GC.

		solvents in g/L			
**short-ID**	**organism**	**acetone**	**ethanol**	**butanol**	**total**
002	*C. beijerinckii* NCIMB 8052	1.45	0.08	5.12	6.64
006	*C. saccharobutylicum* DSM 13864	0.03	0.00	4.20	0.03
007	*C. saccharoperbutylacetonicum* DSM 14923	0.54	0.05	1.43	2.02
041	*C. saccharobutylicum* NCP 262	2.44	0.14	5.04	7.63
123	*C. beijerinckii*	0.07	0.08	0.09	0.25
125	*C. saccharobutylicum*	0.10	2.49	5.74	8.33
126	*C. beijerinckii*	0.10	1.93	4.64	6.67
127	*C. beijerinckii*	1.59	0.08	5.48	7.15
129	*C. diolis* DSM 15410	1.59	0.08	5.10	6.76
131	*C. saccharoperbutylacetonicum*	2.92	0.12	2.63	5.67

Figure S1: GM with different amounts of wheat red dog. Pictures were taken after autoclaving.

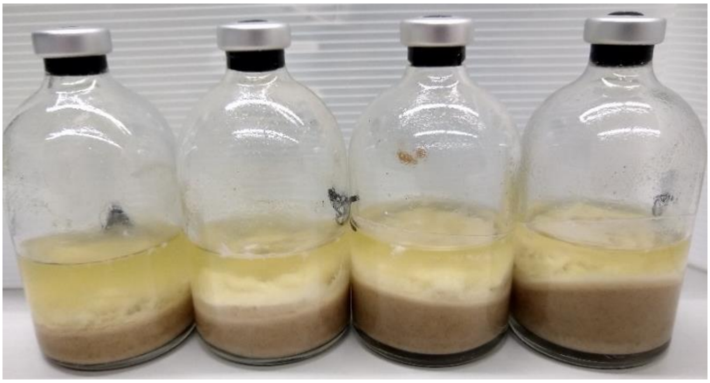

7.2 % 9.9 % 12.3 % 14.7 %

wheat red dog

Figure S2: TLC of enzyme pretreated wheat middlings. 1 % (w/v) wheat middlings were enzymatically macerated with three commercially available enzyme mixtures. The reaction mixture was incubated for 24 h at 50 °C. Xylo-oligosaccharides (xylotriose, xylotetrose and xylopentose; xylo-oligos), D-xylose and D-glucose were used as references. Additional lanes were digitally removed.

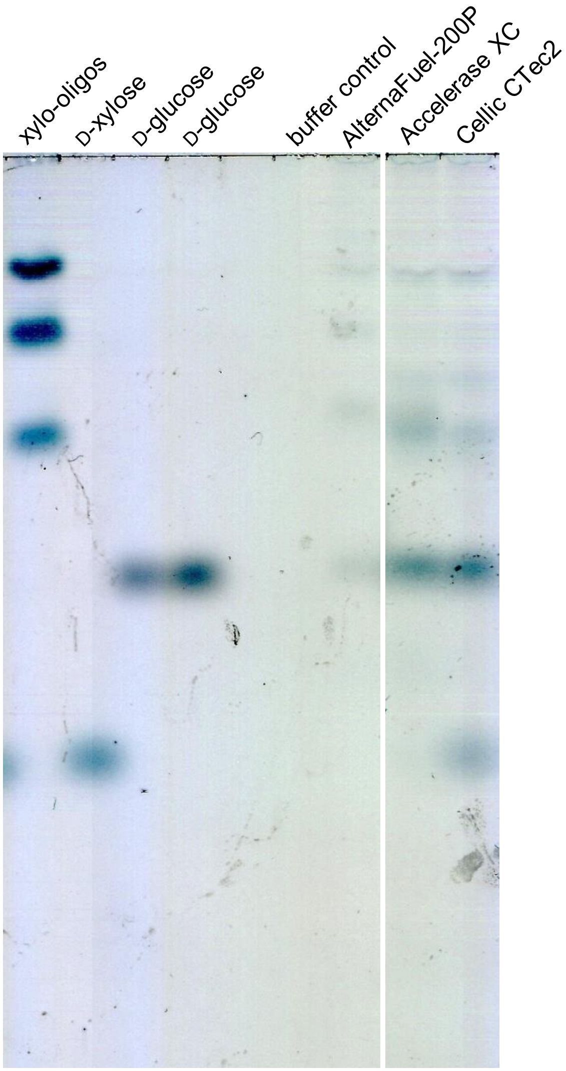

Supplement: Supplementary file 1 — (PDF 356 kb) [file 253_2020_10882_MOESM1_ESM.pdf]
